# Histamine targets myeloid-derived suppressor cells and improves the anti-tumor efficacy of PD-1/PD-L1 checkpoint blockade

**DOI:** 10.1007/s00262-018-2253-6

**Published:** 2018-10-12

**Authors:** Hanna Grauers Wiktorin, Malin S. Nilsson, Roberta Kiffin, Frida Ewald Sander, Brianna Lenox, Anna Rydström, Kristoffer Hellstrand, Anna Martner

**Affiliations:** TIMM Laboratory, Sahlgrenska Cancer Center, University of Gothenburg, Medicinaregatan 1F, Box 425, 413 90 Göteborg, Sweden

**Keywords:** Myeloid-derived suppressor cells, Histamine dihydrochloride, NOX2, Reactive oxygen species, PD-1, Checkpoint inhibition

## Abstract

Myeloid-derived suppressor cells (MDSCs) are immature monocytes and granulocytes that impede immune-mediated clearance of malignant cells by multiple mechanisms, including the formation of immunosuppressive reactive oxygen species (ROS) via the myeloid cell NADPH oxidase (NOX2). Histamine dihydrochloride (HDC), a NOX2 inhibitor, exerts anti-cancer efficacy in experimental tumor models but the detailed mechanisms are insufficiently understood. To determine effects of HDC on the MDSC compartment we utilized three murine cancer models known to entail accumulation of MDSC, i.e. EL-4 lymphoma, MC-38 colorectal carcinoma, and 4T1 mammary carcinoma. In vivo treatment with HDC delayed EL-4 and 4T1 tumor growth and reduced the ROS formation by intratumoral MDSCs. HDC treatment of EL-4 bearing mice also reduced the accumulation of intratumoral MDSCs and reduced MDSC-induced suppression of T cells ex vivo. Experiments using GR1-depleted and *Nox2* knock out mice supported that the anti-tumor efficacy of HDC required presence of NOX2^+^ GR1^+^ cells in vivo. In addition, treatment with HDC enhanced the anti-tumor efficacy of programmed cell death receptor 1 (PD-1) and PD-1 ligand checkpoint blockade in EL-4- and MC-38-bearing mice. Immunomodulatory effects of a HDC-containing regimen on MDSCs were further analyzed in a phase IV trial (Re:Mission Trial, ClinicalTrials.gov; NCT01347996) where patients with acute myeloid leukemia received HDC in conjunction with low-dose IL-2 (HDC/IL-2) for relapse prevention. Peripheral CD14^+^HLA-DR^−/low^ MDSCs (M-MDSCs) were reduced during cycles of HDC/IL-2 therapy and a pronounced reduction of M-MDSCs during HDC/IL-2 treatment heralded favorable clinical outcome. We propose that anti-tumor properties of HDC may comprise the targeting of MDSCs.

**Electronic supplementary material:**

The online version of this article (10.1007/s00262-018-2253-6) contains supplementary material, which is available to authorized users.

## Introduction

Immature myeloid cells (IMCs) accumulate in peripheral organs and in the tumor microenvironment in human and experimental cancer. IMCs normally differentiate into mature myeloid cells such as macrophages, dendritic cells (DCs), and granulocytes upon migration from the bone marrow (BM) to the periphery. This differentiation is frequently defective in cancer with ensuing expansion of IMCs, presumably as the result of the formation of differentiation-inhibitory factors by malignant cells. IMCs may be further activated to acquire immunosuppressive properties by factors produced by activated T cells and tumor stroma cells. These immature immunosuppressive cells are denoted myeloid-derived suppressor cells (MDSCs) [1].

Reactive oxygen species (ROS) are short-lived compounds that arise from electron transfer across biological membranes to form superoxide anion (O_2_^−^) from molecular oxygen. ROS comprise oxygen radicals such as O_2_^−^ and hydroxyl radicals (^**·**^OH) along with non-radicals, including hydrogen peroxide. ROS, formed by the myeloid cell NADPH oxidase (NOX2), are pivotal mediators in the defense against microorganisms [2]. When released into the extracellular space ROS may also trigger dysfunction and apoptosis in neighboring cells, including lymphocytes [3]. This pathway of immunosuppression is exploited by MDSCs, which show increased ROS production by virtue of up-regulated NOX2 activity. In the absence of functional NOX2, MDSCs are less prone to suppress T cells and instead differentiate into macrophages and DCs [4].

Human and murine MDSCs occur in granulocytic (G-MDSCs) and monocytic (M-MDSCs) forms [5]. Phenotypically, human G-MDSCs share the surface markers of neutrophils but differ in buoyant density. Human M-MDSCs are phenotypically distinguished from normal monocytes by their expression density of HLA-DR, where monocytes are CD14^+^HLA-DR^high^ whereas M-MDSCs are CD14^+^HLA-DR^−/low^ [6]. Human M-MDSCs as well as G-MDSCs reportedly produce NOX2-derived ROS and suppress T cell functions in a ROS-dependent manner [7]. Murine MDSCs express GR1 and CD11b, and the murine G-MDSC and M-MDSC subsets are distinguished by their expression of the GR1 epitopes Ly6G and Ly6C. Hence, G-MDSCs are CD11b^+^Ly6G^+^Ly6C^low^, whereas M-MDSCs are CD11b^+^Ly6G^−^Ly6C^high^ [8]. In mice, the capacity to suppress T cells via ROS production is largely confined to the G-MDSC subset [9], whereas murine M-MDSCs rely on nitric oxide synthase (iNOS) for their immunosuppressive properties [1].

The presence of MDSCs is assumed to facilitate the growth and spread of tumors and may also dampen the efficacy of cancer immunotherapies [10]. Several approaches to target MDSCs have been proposed, including blocking the recruitment of MDSCs to the tumor microenvironment [11], eliminating MDSCs [12], targeting their immunosuppressive features [13] or facilitating their maturation [14]. Histamine is a pleiotropic biogenic amine stored in mast cells and basophilic leukocytes [15]. We recently reported that the administration of histamine dihydrochloride (HDC), a histamine salt that dissociates into histamine in solution, promotes the development of monocyte-derived DCs in vitro and in vivo and that these pro-differentiating properties were mediated by inhibition of NOX2 [16]. In addition, Yang et al. showed that mice that lack the histamine-forming histidine decarboxylase, with ensuing histamine deficiency in tissues, are highly susceptible to chemically induced cancer. These histamine-deficient mice were reported to accumulate MDSCs to a higher extent than their wild-type counterparts during the progression of solid tumors [17].

Beyond its purported role in myelopoiesis, HDC inhibits ROS production by myeloid cells in a NOX2-dependent manner and thus reduces the immunosuppressive features of various NOX2^+^ myeloid cells [3]. HDC is approved in Europe, in conjunction with low-dose IL-2, for relapse prevention in patients with acute myeloid leukemia (AML) who have achieved complete remission (CR) after chemotherapy [18]. While details regarding the anti-leukemic action of the HDC component remain to be determined, it has been proposed that HDC targets NOX2-derived immunosuppressive ROS to protect anti-tumor lymphocytes from ROS-induced inactivation [18].

The present study aimed at determining effects of HDC on MDSCs in three murine tumor models known to entail pronounced MDSC accumulation. We report that the systemic administration of HDC, by targeting NOX2, rendered intratumoral MDSCs less immunosuppressive and delayed the growth of murine EL-4 lymphoma and 4T1 breast cancer and, also, that these properties of HDC translated into improved anti-tumor efficacy of antibodies against the programmed cell death receptor 1 (PD-1) and the PD-1 ligand (PD-L1) in EL-4- and MC-38-bearing mice. In addition, the administration of HDC/IL-2 to AML patients in CR was associated with reduced counts of M-MDSCs in blood, which predicted reduced risk of leukemic relapse. We hypothesize that anti-tumor effects of HDC may involve the targeting of MDSCs.

## Materials and methods

### Culture of EL-4, 4T1 and MC-38 cells

The EL-4 and 4T1 cell lines were maintained in RPMI 1640 (VWR, Stockholm, Sweden) and the MC-38 cell line in DMEM without sodium pyruvate (Sigma-Aldrich, St. Louis, MO, USA). Medium was supplemented with 10% fetal calf serum (FCS), 100 µg/ml penicillin, 100 µg/ml streptomycin and 2 mM l-glutamine (EL-4 and 4T1 cells) at 37 °C and 5% CO_2_. Adherent 4T1 and MC-38 cells were detached by trypsinization before expansion. Cells were cultured in vitro for 1–2 weeks prior to inoculation into mice.

### Tumor cell proliferation assay

EL-4 and MC-38 cells were stained with CellTraceViolet Proliferation Kit (Invitrogen, Carlsbad, CA, USA) according to the manufacturer’s instructions. The cells were cultured in the presence or absence of 100 µM HDC (Sigma-Aldrich) for 1–4 days following assessment of proliferation using a four-laser BD LSRFortessa (405, 488, 532, and 640 nm from BD Biosciences, San Diego, CA, USA) and analyzed using FACSDiva software (version 6 or later; BD Biosciences).

The 4T1 cells were cultured for 5 days in the presence or absence of 100 µM HDC. At 30 min or 8 h prior to collection of cells, BrDU at a final concentration of 10 µM was added to the medium. The cells were then fixed, permeabilized, incubated with DNase A, and analyzed on a BD LSRFortessa for BrdU incorporation using the BD Pharmingen BrdU Flow Kit (BD Biosciences).

### EL-4, 4T1 and MC-38 models

Six- to eight-week-old female C57BL/6J and BALB/c mice were obtained from Charles River (Charles River Laboratories, Sulzfeld, Germany). B6.129S6-Cybb^tm1Din^ [*Nox2*-knock out (KO)] mice were originally obtained from Jackson Laboratory (Bar Harbor, ME, USA) and bred in-house. C57BL/6J mice and *Nox2*-KO mice were injected subcutaneously (s.c.) with 1.75–3 × 10^5^ EL-4 cells or 5–10 × 10^5^ MC-38 cells. BALB/c mice were injected s.c. with 4 × 10^5^ 4T1 cells. Mice were treated by intraperitoneal (i.p.) injections of HDC at 1500 µg/mouse (EL-4- and MC-38-bearing mice) or 1000 µg/mouse (4T1-bearing mice) three times per week starting 1 day before tumor inoculation, or with i.p. injections of a mixture of antibodies against PD-1 (α-PD-1; 100–240 µg/mouse; RMP1-14; Nordic Biosite, Stockholm, Sweden) and PD-L1 (α-PD-L1; 100–240 µg/mouse; 10F.9G2; Nordic Biosite) 3, 6 and 10 days after tumor inoculation, or with the addition of HDC to the regimen of PD-1/α-PD-L1 antibodies. Effects of HDC on EL-4 tumor growth confirm and extend a previous study [16]. In some experiments, EL-4 cells were treated with 100 µM HDC in vitro for 3–5 days prior to tumor inoculation. Mice inoculated with in vitro HDC-treated cells did not receive further in vivo treatment. GR1^+^ cells depletion in EL-4-bearing mice was achieved by i.p. injections of GR1-neutralizing antibodies (250 µg, RB6-8C5, BioXcell, West Lebanon, USA) every other day starting once tumors became palpable.

The size of tumors was measured three times per week as the length × width. When several experiments were analyzed, the tumor size was normalized against the mean tumor size of untreated WT control mice, untreated GR1 depleted WT mice, or untreated *Nox2*-KO control mice at the termination of each experiment. Mice were sacrificed and tumors and spleens harvested 2–3 weeks after tumor cell inoculation when the size of the largest tumors had reached a diameter of 1–1.5 cm.

### Processing of spleens, tumors and BM

Single cell suspensions of tumors were prepared by enzymatic digestion using a Tumor Dissociation Kit (Miltenyi Biotec, Lund, Sweden) along with mechanical dissociation utilizing a gentleMACS Dissociator (Miltenyi Biotec) according to the manufacturer’s instructions. BM cells were isolated from femur and tibia of tumor-free naïve mice by crushing the bones using a mortar. BM cells were rinsed and spleens were mashed through a 70-µm strainer and depleted of erythrocytes by Red Blood Cell Lysis buffer (Sigma-Aldrich).

### Flow cytometry analysis of murine samples

Single cell suspensions from tumors and spleens were incubated for 5 min with F_c_-block (BD Biosciences) and then stained with either a myeloid panel of antibodies comprising CD45-BV786 (Clone 30-F11, BD Biosciences), GR1-PE (Clone RB6-8C5, BD Biosciences), CD11b-BV711 (Clone MI/70, BD Biosciences), Ly6C-PerCpCy5.5 (Clone AL-21, BD Biosciences), Ly6G-FITC (Clone IA8, BD Biosciences) and DAPI (Invitrogen) or a lymphoid panel of antibodies comprising CD45-AlexaFlur700 (Clone 30-F11, BD Biosciences), CD3-PE (Clone 145-2C11, eBioscience), NKp46-PE-Cy7 (Clone 29A1.4, eBioscience), CD4-APC (Miltenyi Biotec), CD8-FITC (Miltenyi Biotec), CD44-BV711 (Clone IM7, BD Biosciences), CD62L-BV786 (Clone MEL-14, BD Biosciences), PD-1-BV605 (Clone J43, BD Biosciences) and DAPI (Invitrogen). In some experiments MDCSs were also analyzed for iNOS-PE (Clone CXNFT, eBioscience) expression. Cells were acquired on a BD LSRFortessa and analyzed using FACSDiva.

### T cell suppression assay

GR1^+^ cells were isolated from spleens of EL-4-bearing mice and from BM of tumor-free naïve mice. Single cell suspensions were stained with a Ly6G/C-biotin antibody (clone RB6-8C5, BD Biosciences) followed by incubation with streptavidin-conjugated magnetic beads and positively selected by use of a MACS magnet (Miltenyi Biotech) according to the manufacturer’s instructions. The purity was consistently > 80%. The purified GR1^+^ cells expressed CD11b {98% ± 0.48, [mean ± standard error of the mean (SEM)], *n* = 6}. Splenocytes from OT-1 mice (Rag2/OT-1, Taconic, USA) were stained with CellTraceViolet Proliferation Kit (Invitrogen) according to the manufacturer’s instructions. CellTraceViolet^+^ OT-1 splenocytes were cultured at a 1:1 ratio with GR1^+^ cells from EL-4-bearing or naïve mice in the presence of 10 µg/ml the OT-1 T cell specific peptide SIINFEKL (Sigma-Aldrich) or the control peptide gp100 IMDQVPFSV (AnaSpec, Fremont, USA). The cells were cultured for 3 days in RPM1 1640 supplemented with 10% FCS, 100 µg/ml penicillin, 100 µg/ml streptomycin and 2 mM l-glutamine at 37 °C and 5% CO_2_ and thereafter stained with FITC-anti-CD8 (Miltenyi Biotec) before measuring T cell proliferation by flow cytometry. Results were analyzed with FlowJo Version 10.1 (TreeStar, Ashland, USA).

### Generation of human monocyte-derived MDSCs

PBMCs were prepared from healthy blood donor buffy coats by Ficoll-Paque (Lymphoprep, Nycomed, Oslo, Norway) density centrifugation. Monocytes were isolated by adherence and cultured in Iscoves’ medium supplemented with 10% human AB serum, 2 mM l-glutamine, 100 µg/ml penicillin, 100 µg/ml streptomycin, 1 ng/ml interleukin 6 (hIL-6, Sigma-Aldrich) and 10 ng/ml granulocyte macrophage colony-stimulating factor (hGM-CSF, Peprotech, Rocky Hill, USA) in the presence or absence of 100 µM HDC. In control experiments, adherent monocytes were cultured in the absence of cytokines. One-half of the medium was replaced and HDC was again added after 2 days of culture. Cells were examined for expression of HLA-DR (antibody: HLA-DR-APC-Cy7, Clone C243, BD Biosciences) by flow cytometry after 5 days of culture.

### Detection of ROS by chemiluminescence

Superoxide anion production in response to the hexapeptide Typ-Lys-Tyr-Met-Val-d-Met (d-peptide, R&D Systems, Minneapolis, MN, USA) or *N*-formyl-Met-Leu-Phe (fMLF, Sigma-Aldrich) by murine cells from tumors and spleens or by human cytokine-induced MDSCs was measured by isoluminol chemiluminescence (CL) as described [19]. Results are presented as curves displaying continuous ROS formation or as the area under the curve normalized to the mean area under the curve for cells from tumor-bearing control mice.

### MDSCs in a clinical trial of HDC/IL-2

In a phase IV trial (Re:Mission; ClinicalTrials.gov; NCT01347996), 84 adult patients with AML in first complete remission received ten consecutive 21-day cycles of HDC and interleukin-2 (HDC/IL-2) for 18 months or until relapse or death. The trial is described in detail elsewhere [20, 21]. Blood was collected before and after the first and third HDC/IL-2 treatment cycle. PBMCs were isolated and cryopreserved at local sites and shipped on dry ice to the central laboratory at the Sahlgrenska Cancer Center, University of Gothenburg, Sweden.

PBMCs were stained, as described in [20], with a panel of antibodies against myeloid cells to determine the content of MDSCs in blood. The MDSC-panel included the following antibodies from BD Biosciences: CD3-PerCpCy5.5 (clone HIT3A), CD19-PerCPCy5.5 (SJ25C1), CD16-Brilliant Violet 605 (3G8), HLA-DR-APCH7 (G46-6), CD14-PECy7 (MφP9) and CD56-PerCp eflour 710 from CMSSB, Thermo Fischer Scientific, USA. Stained samples were acquired on a BD FACSAria. PBMCs were also stained using a second panel to determine the expression level of H_2_R and gp91^phox^ (the catalytic subunit of NOX2) on MDSCs using the following stains and antibodies: LIVE/DEAD fixable yellow stain (Life Technologies, Grand Island, NY, USA), CD33-PECy7 (P67.6), CD16-APC-H7 (3G8), HLA-DR-Qdot605 (G46-6) (all from BD Biosciences), CD14-Qdot655 (TüK4, Life Technologies), anti-histamine H_2_ receptor (polyclonal rabbit IgG, MBL International, Woburn, MA, USA), goat anti-rabbit-PerCpCy5.5 and gp91^phox^-FITC (7D5, MBL International). Samples were analyzed on a four-laser BD LSRFortessa flow cytometer and data analysis was performed using FlowJo software, version 7.6.5 or later (TreeStar, AShlandm OR). Sixty-two patients were analyzed using the MDSC-panel and 49 patients were analyzed using the H_2_R and gp91^phox^ panel. The selection of patients for analysis was based on the availability of viably frozen PBMCs. Differential counts, obtained from participating centers, were used to calculate the absolute counts of MDSCs in patient blood.

### Statistics

Statistical analyses were performed using GraphPad Prism software (version 6.0 or later). Paired and unpaired *t* tests were utilized for comparisons between two groups and one and two-way ANOVA followed by Holm–Sidak’s test was used for comparisons between > two groups. In experiments using MC-38 tumor-bearing mice, tumors were completely eradicated by immunotherapy in some animals. In these experiments, the linear mixed effects model was employed to compare the slope of tumor growth curves from day 6 until the experimental endpoint, or until the first size = 0 measurement. For survival analysis, the logrank (Mantel-Cox) test was utilized to compare patients showing a strong or a low/no reduction of MDSCs (dichotomized by the median reduction) during treatment with HDC/IL-2.

## Results

### HDC reduces tumor progression by targeting NOX2^+^ MDSCs

**Fig. 1:**
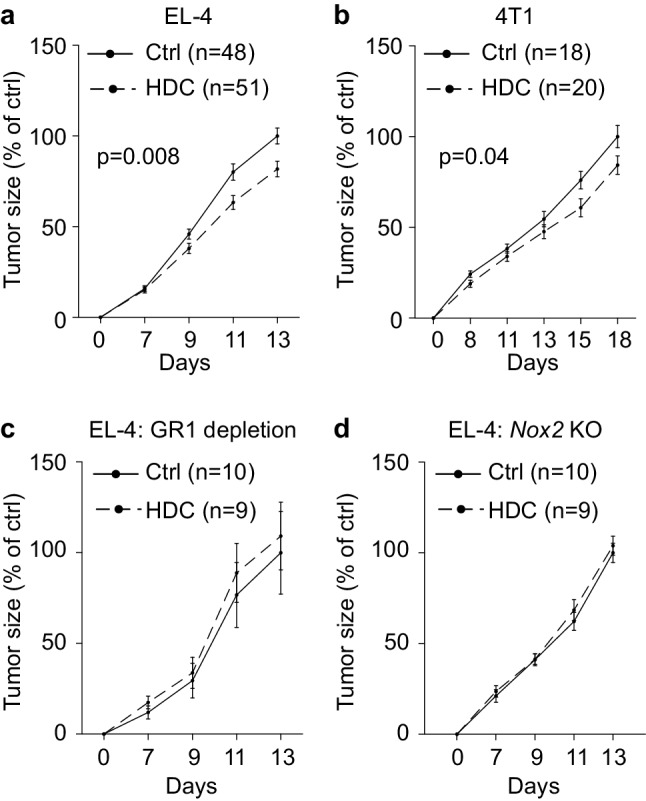
HDC reduces the growth of EL-4 lymphoma and 4T1 mammary carcinoma in mice. Mice were either untreated (Ctrl, solid lines) or treated with HDC (dashed lines) thrice weekly starting 1 day before tumor cell inoculation. **a, b** Growth of **a** EL-4 lymphomas and **b** 4T1 tumors in wild-type mice. **c** EL-4 growth in wild-type mice depleted of GR1^+^ cells. **d** EL-4 tumor growth in *Nox2*-KO mice. The tumor size was normalized against the mean tumor size of control mice at the end of each experiment, i.e. against untreated WT mice in **a, b**, untreated GR1-depleted WT mice in **c**, and untreated *Nox2*-KO mice in **d**. Results were analyzed using two-way ANOVA

In agreement with a previous report [16], the systemic administration of HDC significantly reduced the in vivo growth of EL-4 lymphomas (Fig. 1a). HDC also reduced the growth of 4T1 mammary carcinoma (Fig. 1b) with a similar, albeit non-significant, trend observed in MC-38-bearing mice (Supplementary Fig. 1a). To elucidate the role of MDSCs for the anti-tumor efficacy of HDC, mice inoculated with EL-4 lymphoma cells were depleted of GR1^+^ cells using the GR1-neutralizing antibody RB6-8C5. As determined by FACS analysis at the end of the experiment, intratumoral GR1^+^CD11b^+^ MDSCs were reduced by approximately 75% following GR1 antibody treatment (Supplementary Fig. 2a). In GR1-depleted animals, treatment with HDC did not affect EL-4 lymphoma growth (Fig. 1c) but significantly reduced lymphoma growth in simultaneously performed experiments in non-GR1-depleted animals (*p* = 0.03 at day 10, Students’ *t* test, Supplementary Fig. 2b). In agreement with a previous report [22] treatment with GR1-neutralizing antibodies per se did not significantly impact on EL-4 lymphoma growth (Supplementary Fig. 2b).

The effect of HDC treatment on EL-4 lymphoma growth was also evaluated in *Nox2*-KO mice, where MDSCs accumulate but do not generate NOX2-derived ROS. HDC did not alter lymphoma growth in the *Nox2*-KO mice (Fig. 1d). HDC did not affect the proliferation or cell cycling of EL-4 or 4T1 cells but slightly reduced the proliferation of MC-38 cells after two days in culture (Supplementary Fig. 1b–f). In vivo growth of EL-4 cells was not affected by previous in vitro exposure to HDC (Supplementary Fig. 1g).

### Effects of HDC on myeloid and lymphoid populations in tumor-bearing mice

**Fig. 2:**
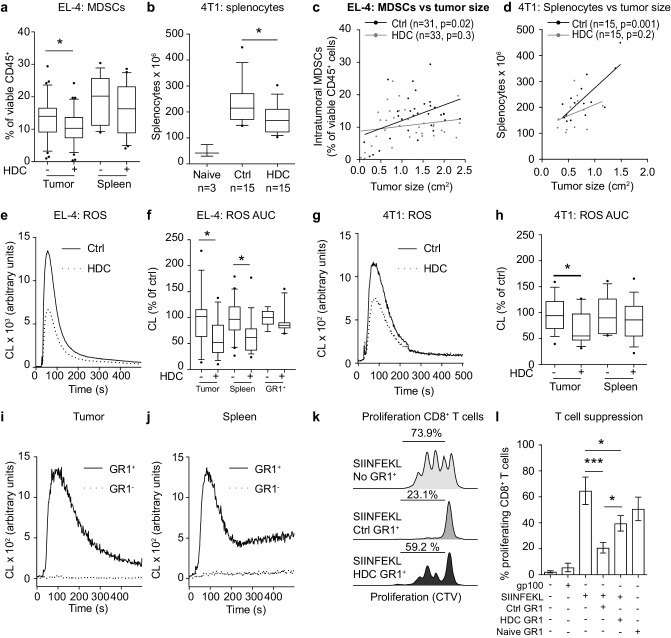
HDC reduces the immunosuppressive properties of MDSCs in mice carrying EL-4 and 4T1 tumors. EL-4-bearing mice were euthanized after 2 weeks and 4T1-bearing mice after 3 weeks of tumor growth when the mean tumor size of untreated mice reached approximately 1.5 cm^2^. **a** Accumulation of intratumoral and splenic MDSCs in EL-4-bearing mice. Content of MDSCs was examined in control mice (*n* = 31 for intratumoral MDSCs, *n* = 19 for splenic MDSCs) and in HDC-treated mice (*n* = 33 for intratumoral MDSCs, *n* = 21 for splenic MDSCs). **b** Counts of splenocytes in tumor-free (naïve) and control or HDC-treated 4T1-bearing mice. Correlation between **c** intratumoral MDSCs and tumor size in EL-4-bearing mice or **d** splenocytes and tumor size in 4T1-bearing mice in control (black) and HDC-treated (grey) animals. **e** Mean d-peptide-induced ROS production from leukocytes recovered from tumors of control (solid line, *n* = 18) and HDC-treated (HDC, dotted line, *n* = 17) EL-4-bearing mice. **f** ROS formation (area under the curve) in response to d-peptide by single cell suspensions from tumors, spleens or splenocyte-derived GR1^+^ cells isolated from control (tumor *n* = 18, spleen *n* = 20, GR1^+^*n* = 9) or HDC-treated (tumor *n* = 17, spleen *n* = 19, GR1^+^*n* = 11) EL-4-bearing mice. **g** Mean d-peptide-induced ROS production from leukocytes recovered from tumors of control (solid line, *n* = 15) and HDC-treated (HDC, dotted line, *n* = 14) 4T1-bearing mice. **h** ROS formation (area under the curve) in response to d-peptide stimulation by single cell suspensions from tumors or spleens isolated from control (tumor *n* = 15, spleen *n* = 15) or HDC-treated (tumor *n* = 15, spleen *n* = 15) 4T1-bearing mice. In **f, h** ROS formation was normalized against the mean ROS formation of tumor-bearing control mice in each experiment. **i, j** ROS formation in response to d-peptide from GR1^+^ (solid line, *n* = 3) and GR1^−^ (dotted line, *n* = 3) cells isolated from **i** tumors and **j** spleens of control EL-4-bearing mice. **k, l** Proliferation of OT-1 CD8^+^ T cells was determined after 3 days of culture. **k** Representative histograms of CellTraceViolet-stained SIINFEKL-stimulated OT-1 CD8^+^ splenocytes in the absence of GR1^+^ cells (SIINFEKL, No GR1^+^) or in the presence of GR1^+^ cells isolated from spleens of control or HDC-treated EL-4-bearing mice. **l** Percentage of proliferating CD8^+^ T cells in the absence of stimuli (*n* = 3), in response to a control peptide (gp100, *n* = 3) or in response to an OT-1 specific peptide (SIINFEKL, *n* = 3). In specified wells, GR1^+^ cells that had been isolated from control (*n* = 5) or HDC-treated (*n* = 6) EL-4-bearing mice or GR1^+^ cells isolated from tumor-free mice (*n* = 2) were present at a 1:1 ratio with the SIINFEKL stimulated OT-1 splenocytes during the course of proliferation. Statistical differences were evaluated using Student’s *t* test or one-way ANOVA. Linear regression was utilized to analyze correlations. *p < 0.05, ***p < 0.001

In accordance with a previous report [9], EL-4 and 4T1 growth was associated with a pronounced increase of MDSCs in tumors and spleens (Fig. 2a and Supplementary Fig. 3a). Treatment of mice with HDC significantly reduced the accumulation of MDSCs within EL-4 lymphomas, but not in spleen (Fig. 2a). Mice inoculated with 4T1 cells acquired enlarged spleens where approximately 50% [52% ± 2.5 (mean ± SEM), *n* = 30] of splenocytes were MDSCs. Treatment of mice with HDC reduced the number of splenocytes but did not alter the content of MDSCs in tumors or spleens in this model (Fig. 2b and Supplementary Fig. 3a). The vast majority of MDSCs in tumor-bearing mice were G-MDSCs. HDC did not affect the distribution of MDSC subtypes in EL-4-bearing mice (Supplementary Fig. 3b) but significantly reduced the accumulation of splenic and tumor-infiltrating M-MDSCs in 4T1-bearing mice (Supplementary Fig. 3c).

We did not observe a significant increase in the number of tumor-infiltrating or splenic CD8^+^ T cells in HDC-treated EL-4 or 4T1-bearing mice (Supplementary Fig. 4a, b). However, a negative correlation was noted between the percentage of intratumoral MDSCs and tumor-infiltrating CD8^+^ T cells in both models (Supplementary Fig. 4c, d). Treatment of mice with HDC significantly enhanced the proportion of CD62L^−^ cells (comprising CD44^+^ effector memory and CD44^−^ effector populations) among CD8^+^ T cells in EL-4-bearing mice with a similar trend in the 4T1 model (Supplementary Fig. 4e, f). Treatment of EL-4-bearing mice with HDC did not significantly alter the percentage of intratumoral CD4^+^ T cells, NK cells or B cells but slightly reduced the percentage of CD4^+^ T cells and NK cells in spleens (Supplementary Fig. 4g–i).

### HDC reduces NOX2-dependent immunosuppression in MDSCs

To determine whether the reduction in MDSCs and splenocytes following HDC treatment was secondary to the reduced tumor size we correlated the tumor size with MDSC content in the EL-4 model and with the number of splenocytes in the 4T1 model. A positive correlation was noted between the size of tumors and the percentage of intratumoral MDSCs in EL-4-bearing control mice and with splenocytes in 4T1-bearing control mice (Fig. 2c, d). No such correlations were observed in HDC-treated EL-4- or 4T1-bearing mice (Fig. 2c, d), suggesting that MDSCs accumulating in tumors following HDC treatment might be less immunosuppressive.

We observed that in vivo administration of HDC reduced the capacity of leukocytes isolated from EL-4 and 4T1 tumors as well as from splenocytes of EL-4-bearing mice to generate NOX2-derived ROS, with a similar trend observed for isolated GR1^+^ cells (Fig. 2e–h). Leukocytes recovered from tumors and spleens of EL-4-bearing control mice were separated into GR1^+^ and GR1^−^ fractions and analyzed for ROS-forming capacity. The results confirmed that ROS production was confined to the GR1^+^ MDSC population (Fig. 2i, j). We also observed that GR1^+^ cells recovered from EL-4-bearing control mice strongly suppressed T cell proliferation and were significantly more suppressive than MDSCs recovered from HDC-treated mice (Fig. 2k, l). MDSCs may also exert immunosuppression via additional mechanisms, including iNOS-derived NO production [6]. We found no significant difference in iNOS expression, as determined by flow cytometry, between MDSCs isolated form HDC-treated and control EL-4 bearing mice (*p* = 0.24, *n* = 4, Students’ *t* test).

### HDC reduces the in vitro generation of human MDSC-like cells

**Fig. 3:**
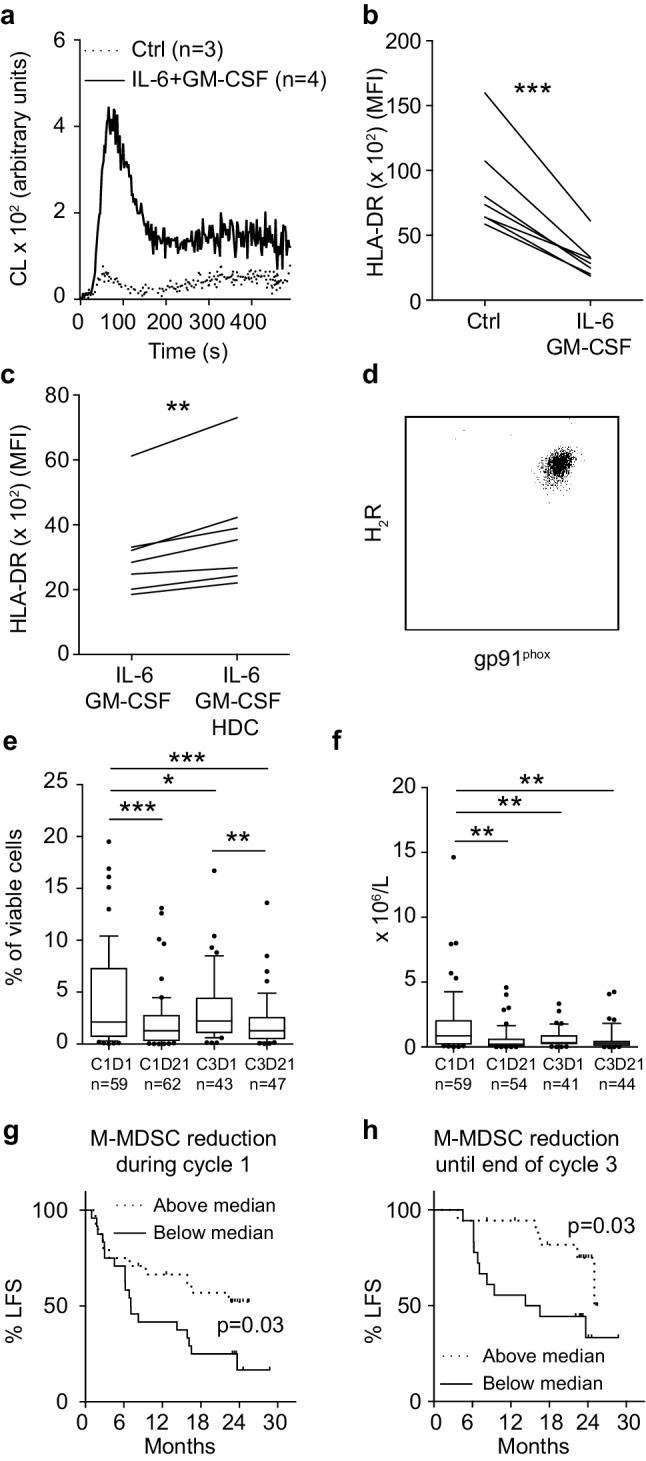
HDC targets human MDSCs in vitro and in vivo. **a–c** Human monocytes were cultured in the absence of stimuli or in the presence of IL-6 and GM-CSF for 5 days to induce MDSC-like cells. **a** ROS production from cultured monocytes (ctrl, dotted line) and MDSC-like cells (IL-6 + GM-CSF, solid line) in response to stimulation with fMLF. **b** Expression of HLA-DR on monocytes after 5 days of culture in absence of stimuli (Ctrl) and in presence of IL-6 and GM-CSF (*n* = 7). **c** Expression of HLA-DR on monocytes cultured for 5 days with IL-6 and GM-CSF in the absence or presence of 100 µM HDC (*n* = 7). **d–h** AML patients in CR received HDC/IL-2 immunotherapy in 3-week cycles. **d** Expression of H_2_R and gp91^phox^ on M-MDSCs for a representative patient. **e** Frequency and **f** the absolute counts of M-MDSCs before (cycle 1, day 1; C1D1) and after the first treatment cycle (cycle 1, day 21; C1D21) and at the beginning (cycle 3, day 1; C3D1) and end (cycle 3, day 21; C3D21) of the third treatment cycle. **g, h** Impact of M-MDSC reduction on leukemia-free survival (LFS) in patients undergoing HDC/IL-2 therapy. Patients were dichotomized by **g** the median reduction of M-MDSC counts during the first treatment cycle (*n* = 48), and **h** the median reduction of M-MDSCs from the start of cycle 1 to the end of cycle 3 (*n* = 36). Results were analyzed by Student’s paired *t* test or by the log rank test. *p < 0.05, **p < 0.01, ***p < 0.001

HDC was previously shown to facilitate the maturation of human and murine myeloid cells [16, 17]. We, therefore, determined effects of HDC on the cytokine-induced generation of human MDSCs in vitro. IL-6 and GM-CSF induced an MDSC-like phenotype in monocytes characterized by enhanced production of NOX2-derived ROS in response to fMLF (Fig. 3a) and reduced expression of HLA-DR in all donors (*n* = 12) albeit to a variable degree (10–70% reduction in MFI of HLA-DR). We noted that for donors showing a robust cytokine-induced generation of MDSCs, as determined by a > 50% reduction in monocytic HLA-DR expression (7 out of 12 donors, Fig. 3b), incubation with HDC significantly reduced the cytokine-induced down-regulation of HLA-DR (Fig. 3c).

### Effect of HDC-based immunotherapy on human monocytic MDSCs

We analyzed the content of MDSCs in blood samples from patients with AML, who had been treated with HDC in conjunction with low dose IL-2, to determine effects of treatment with HDC on human MDSCs in vivo. PBMCs from patient blood samples were analyzed for content of M-MDSCs before and after treatment cycle one and three [i.e., cycle 1 day 1 and day 21 (C1D1 and C1D21) and cycle 3 day 1 and day 21 (C3D1 and C3D21)]. The gating strategy from a representative sample is shown in Supplementary Fig. 5. M-MDSCs were found to consistently express high levels of gp91^phox^, the catalytic subunit of NOX2, and H_2_R (Fig. 3d). The frequency and absolute counts of M-MDSCs in blood was significantly reduced during treatment with HDC/IL-2 (Fig. 3e, f). When patients were dichotomized based on above or below median reduction in total number of M-MDSCs within cycle one or between the onset of therapy (C1D1) and the end of cycle three (C3D21), it was observed that a strong reduction in M-MDSC counts significantly predicted leukemia-free survival (Fig. 3g, h).

### HDC enhances the anti-tumor efficacy of α-PD-1 and α-PD-L1 antibodies

**Fig. 4:**
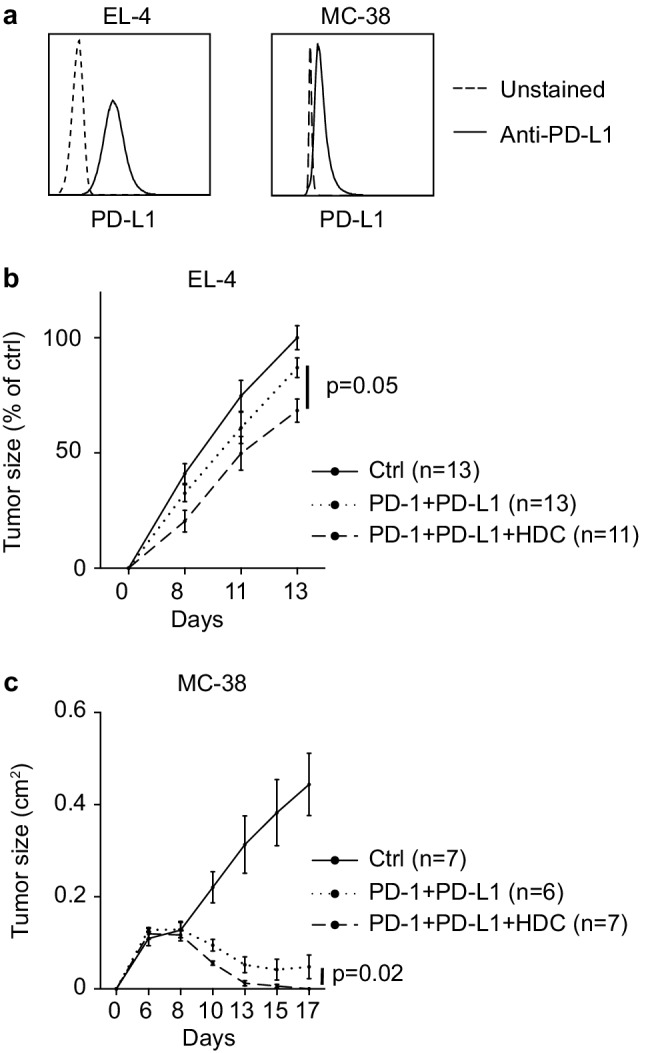
HDC improves the anti-tumor efficacy of α-PD-1/α-PD-L1 immunotherapy. **a** EL-4 and MC-38 cells were stained for expression of PD-L1 (solid line) or were left unstained (dashed line). Growth of **b** EL-4 and **c** MC-38 tumors in control (solid line), α-PD-1/α-PD-L1-treated (dotted line), or HDC/α-PD-1/α-PD-L1-treated (dashed line) mice. In experiments using EL-4 cells, tumor size was normalized against the mean tumor size of control mice at the end of each of four experiments, and results were analyzed using two-way ANOVA. In the MC-38 model, the difference in slope between HDC/α-PD-1/α-PD-L1 and α-PD-1/α-PD-L1 treatment was analyzed by linear mixed models

The finding that HDC may target MDSC-related immunosuppression in humans and mice incited us to investigate if HDC impacted on the efficiency of CD8^+^ T cell-enhancing immunotherapy. EL-4 cells expressed high levels of PD-L1 (Fig. 4a). Also, 77% ± 5.5 (mean ± SEM; *n* = 11) of intratumoral M-MDSCs and 76% ± 2.3 (mean ± SEM; *n* = 11) of intratumoral G-MDSCs expressed PD-L1, and 77% ± 2.8 (mean ± SEM; *n* = 20) of tumor-infiltrating CD8^+^ T cells expressed PD-1 in this model. Treatment of mice with HDC in vivo did not alter the expression of PD-L1 on MDSCs or PD-1 on CD8^+^ T cells (data not shown). Treatment of EL-4-bearing mice with α-PD-1/α-PD-L1 antibodies tended to reduce tumor growth rate. The combination of HDC and α-PD-1/α-PD-L1 was superior to monotherapy with either HDC or α-PD-1/α-PD-L1 in reducing EL-4 tumor growth (Fig. 4b). Analysis of infiltrating immune populations in EL-4 lymphomas showed that α-PD-1/α-PD-L1 treatment did not affect MDSC, T or NK cell accumulation but slightly increased the fraction of CD8^+^ T cells displaying an effector phenotype (Supplementary Fig. 6a–e). The combined regimen of HDC/α-PD-1/α-PD-L1 was also assessed in the 4T1 model. As reported elsewhere [23], 4T1 tumor growth was unaffected by α-PD-1/α-PD-L1 treatment. In this model, the combination of HDC/α-PD-1/α-PD-L1 was not superior to HDC alone in reducing tumor growth (data not shown).

Murine colorectal MC-38 cells expressed PD-L1 (Fig. 4a), and 70% ± 5.5 (mean ± SEM; *n* = 11) of G-MDSCs and 59% ± 3.2 (mean ± SEM; *n* = 11) of M-MDSCs were also PD-L1^+^. The expression of PD-1 was modest in MC-38 tumor-infiltrating CD8^+^ T cells (data not shown). MC-38 tumor growth was nevertheless strongly reduced by treatment with α-PD-1/α-PD-L1; in these mice, tumors expanded during the first week after tumor cell inoculation and then regressed. Treatment with HDC further improved the anti-tumor efficacy of α-PD-1/α-PD-L1 (Fig. 4c). At days 10 and 13, tumor reduction in the HDC/α-PD-1/α-PD-L1 group was superior to treatment with α-PD-1/α-PD-L1 (*p* = 0.01 and 0.04, respectively, two-way ANOVA). At the end of the experiment 50% of mice treated with α-PD-1/α-PD-L1 monotherapy were tumor-free whereas complete tumor clearance was noted in 100% of mice receiving HDC/α-PD-1/α-PD-L1. To enable analysis of MC-38 infiltrating immune populations following immunotherapy, mice were inoculated with a higher number of tumor cells to reduce the likelihood of complete tumor eradication at the experimental endpoint. The added benefit of HDC to α-PD-1/α-PD-L1 therapy was demonstrated also following inoculation of a higher number of MC-38 tumor cells (Supplementary Fig. 7a). In these experiments, treatment of MC-38 tumor-bearing mice with α-PD-1/α-PD-L1 or HDC/α-PD-1/α-PD-L1 tended to increase the fraction of intratumoral CD8^+^ T cells and significantly increased the fraction of CD8^+^ T cell with an effector phenotype (Supplementary Fig. 7b, c). The percentage of intratumoral CD4^+^ T cells was not altered, while a reduction in tumor infiltrating NK cells was noted (Supplementary Fig. 7d, e).

## Discussion

This study aimed at clarifying to what extent the anti-tumor properties of HDC, an inhibitor of NOX2-derived ROS [3], may be mediated by the targeting of MDSCs. Based on results showing that HDC was devoid of anti-tumor efficacy in mice genetically deficient in NOX2 and in mice where MDSCs were depleted by GR1-neutralizing antibodies, we conclude that the anti-tumor properties of HDC rely on the presence of NOX2^+^ GR1^+^ cells. Although alternative or supplementary mechanisms are conceivable, these findings confirm and extend results suggesting that HDC targets NOX2 to exert anti-tumor efficacy in murine cancer models [16, 24, 25]. We also report that treatment of mice with HDC reduced the accumulation of intratumoral MDSCs and the number of splenocytes in two experimental tumor models and that the use of a HDC-based regimen reduced MDSC counts in blood of AML patients in complete remission.

It may be asked if the reduction in the intratumoral content of MDSC and splenomegaly observed after treatment with HDC was secondary to a direct effect of HDC on tumor cells. However, HDC did not affect the in vitro proliferation of EL-4 lymphoma and 4T1 mammary carcinoma cells. Additionally, we did not observe any correlation between tumor growth on the one hand and intratumoral MDSC or splenomegaly on the other in HDC-treated mice, which argues that the reduction of MDSC was not secondary to the reduced tumor size. Instead, the reduction of MDSCs may be explained by pro-differentiating properties of HDC resulting in, i.e., increased numbers of intratumoral DCs [16]. Earlier studies also imply that endogenous histamine is critical for appropriate maturation of myeloid cells [17]. Furthermore, MDSCs isolated from *Nox2*-KO mice more readily differentiate into DCs and macrophages [4]. Our finding that NOX2 inhibition, via HDC, limits cytokine-induced generation of human MDSCs in vitro further supports that ROS may prevent the differentiation of myeloid cells and that HDC may overcome the insufficient differentiation. On a similar note, the administration of All-trans retinoic acid (ATRA) to tumor-bearing mice was shown to reduce MDSC counts in several experimental tumor models and to promote the maturation of MDSCs into DCs and macrophages [14]. The pro-differentiating properties of ATRA were secondary to neutralization of elevated ROS levels in MDSCs [26]. Together with the herein reported results, these findings support that NOX2-derived ROS may be targeted for appropriate myeloid cell maturation.

In agreement with a previous study [27], the number of cells with MDSC-like phenotype was low in AML patients who had achieved CR after receiving chemotherapy. We observed that counts of M-MDSCs in blood were further reduced during the first cycle of HDC/IL-2, and that a strong reduction heralded a favorable course of disease. Since the patients had received chemotherapy approximately 2 months prior to treatment with HDC/IL-2, the distributional changes in myeloid cell populations may, in part, reflect reconstitution of hematopoietic cells. Also, despite that a previous study in renal cell carcinoma patients suggested that monotherapy with IL-2 does not affect the frequency of cells with a MDSC phenotype [28], we cannot rule out that the IL-2 component may have contributed to the observed reduction of MDSCs. With these precautions, our results suggest that treatment with HDC may affect the human MDSC compartment and that MDSCs may constitute a targetable population of relevance to the efficiency of immunotherapy in AML.

In addition to limiting the accumulation of MDSCs in tumor-bearing mice, treatment with HDC reduced the formation of NOX2-derived ROS ex vivo. In line with this finding we observed that MDSCs isolated from HDC-treated mice showed a reduced capacity to suppress CD8^+^ T cell proliferation, thus implying that HDC targets a significant effector function in MDSC-mediated immunosuppression. Notably, treatment of mice with HDC did not improve tumor infiltration of CD8^+^ T cells, despite a positive correlation between accumulation of intratumoral MDSCs and tumor-infiltrating CD8^+^ T cells. Instead, we observed that treatment with HDC was accompanied by the accumulation of intratumoral effector CD8^+^ T cells in EL-4-bearing mice. While further studies are required to define the detailed mechanisms involved, this finding supports that HDC may promote effector functions of tumor-infiltrating CD8^+^ T cells.

It was earlier reported that the targeting of MDSCs by use of colony stimulating factor 1 receptor blockade synergizes with α-PD-1/α-PD-L1 checkpoint inhibition in reducing murine neuroblastoma progression in vivo [11]. These results incited us to investigate if HDC may promote the anti-tumor efficacy of checkpoint inhibition. We observed that HDC enhanced the efficacy of α-PD-1/α-PD-L1 in reducing EL-4 and MC-38 tumor growth. In the EL-4 and MC-38 models, α-PD-1/α-PD-L1 treatment triggered an increased proportion of intratumoral CD8^+^ effector T cells. In the MC-38 model, α-PD-1/α-PD-L1 treatment also tended to increase the presence of CD8^+^ T cells in tumors. In patients, an optimal anti-tumor efficacy of α-PD-1 therapy is generally believed to depend on pre-existing tumor-infiltrating CD8^+^ T cells [29]. The finding that treatment of mice with HDC did not trigger a significant influx of CD8^+^ T cells into tumors suggests that combining HDC and α-PD-1/α-PD-L1 therapy with agents that enhance T cell infiltration, such as chemotherapy or α-VEGF antibodies [30, 31], might further improve anti-tumor efficacy.

In conclusion, our results suggest that in vivo treatment with HDC reduces the accumulation and immunosuppressive features of MDSCs and improves the anti-tumor efficacy of checkpoint blockade in murine EL-4 lymphoma and MC-38 colon carcinoma.

### Electronic supplementary material

Below is the link to the electronic supplementary material.

Supplementary material 1 (PDF 414 KB)

## Abbreviations

AML: Acute myeloid leukemia
ATRA: All-trans retinoic acid
CR: Complete remission
C1D1: Cycle 1, day 1
C1D21: Cycle 1, day 21
C3C1: Cycle 3, day 1
C3D21: Cycle 3, day 21
D-peptide: Typ-Lys-Tyr-Met-Val-d-Met
fMLF: *N*-formyl-Met-Leu-Phe
G-MDSC: Granulocytic myeloid-derived suppressor cells
HDC: Histamine dihydrochloride
H_2_R: Histamine H_2_-type receptor
IMC: Immature myeloid cells
LFS: Leukemia-free survival
MDSC: Myeloid-derived suppressor cells
M-MDSC: Monocytic myeloid-derived suppressor cells
NOX2: NADPH oxidase type 2
O_2_^−^: Superoxide anion
^·^OH: Hydroxyl radical
ROS: Reactive oxygen species
