# Supplementary material for: Histamine targets myeloid-derived suppressor cells and improves the anti-tumor efficacy of PD-1/PD-L1 checkpoint blockade
Source: Cancer Immunol Immunother. 2018 Oct 12;68(2):163–74. doi: 10.1007/s00262-018-2253-6 (PMC6394491; doi:10.1007/s00262-018-2253-6)
Supplement: Supplementary file 1 — Supplementary material 1 (PDF 414 KB) [file 262_2018_2253_MOESM1_ESM.pdf]

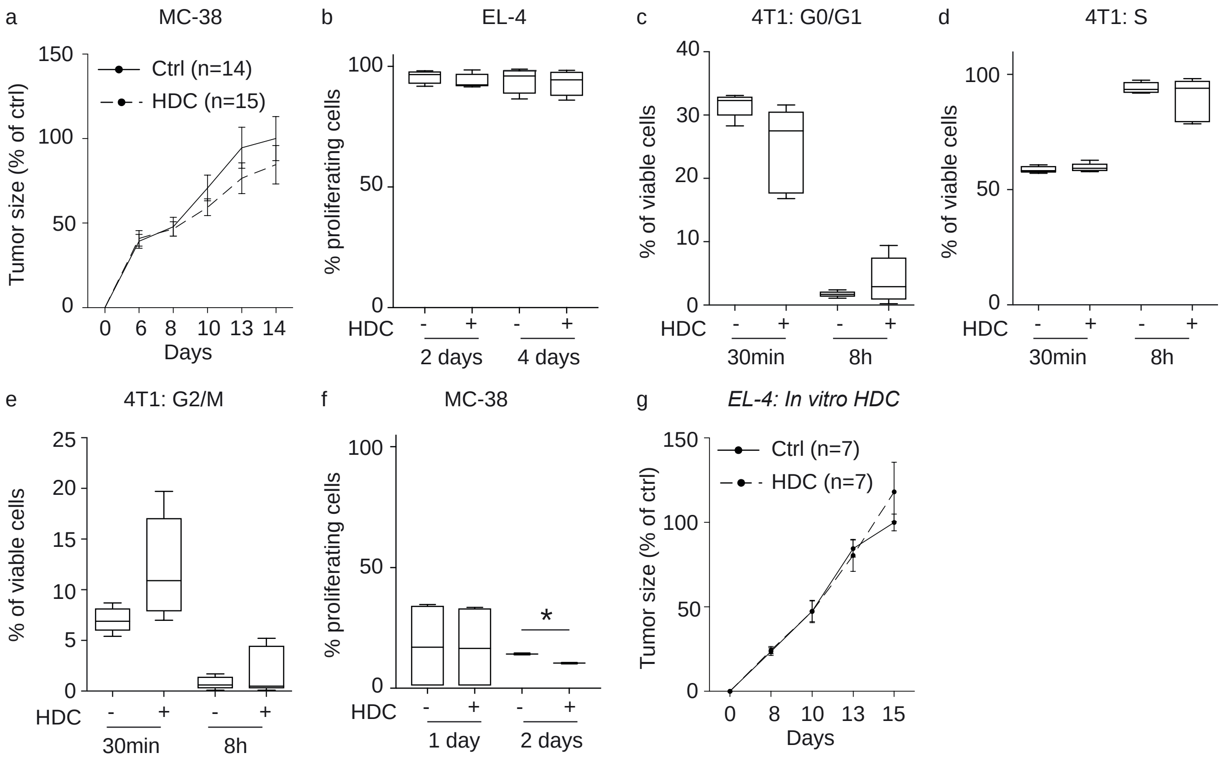

Supplementary figure 1

Effects of HDC on EL-4, 4T1 and MC-38 cells. (**a**) Growth of MC-38 tumors in untreated (solid line) and HDC-treated (dashed line) mice. (**b**) EL-4, (**c-e**) 4T1 and (**f**) MC-38 cells were cultured in the presence or absence of HDC for one to four days (EL-4 and MC-38: n=4, 4T1: n=5) following assessment on proliferation (**b**, **f**) or cell cycle phases (**c-e**). (**g**) S.c. growth of EL-4 tumors that were untreated (solid line) or treated with HDC for three to five days *in vitro* (dashed line) prior to tumor cell inoculation. Statistical differences were analyzed using Student's *t* test. * $p<0.05$

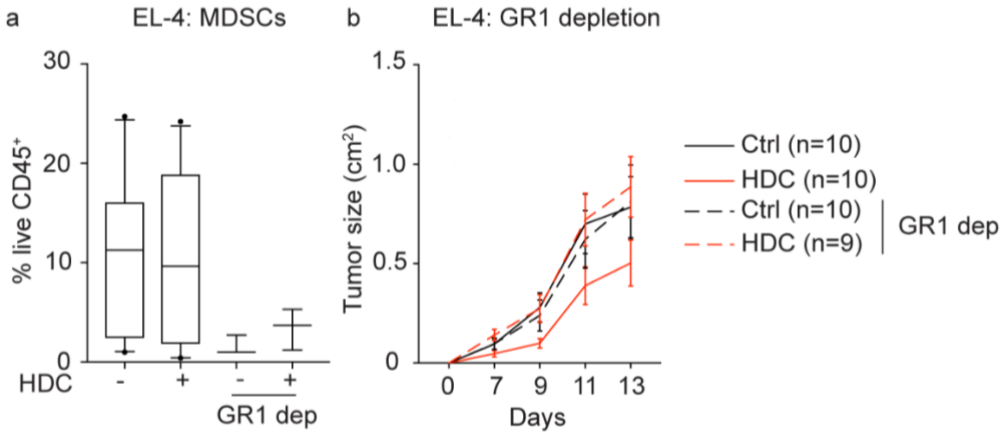

Supplementary figure 2

HDC does not affect tumor growth in GR1-depleted animals. (**a**) Accumulation of intratumoral MDSCs in EL-4-bearing mice with or without treatment with GR1-neutralizing antibodies (GR1 dep) (n=10 for with mice not subjected to anti-GR1 treatment, n=3, for the anti-GR1 treated groups). (**b**) Growth of EL-4 tumors in untreated control mice (Ctrl; black solid line), HDC-treated mice (HDC; red solid line), in GR1-depleted untreated mice (GR1 dep, Ctrl; black dashed line) and HDC treated GR1-depleted mice (GR1 dep, HDC; red dashed line).

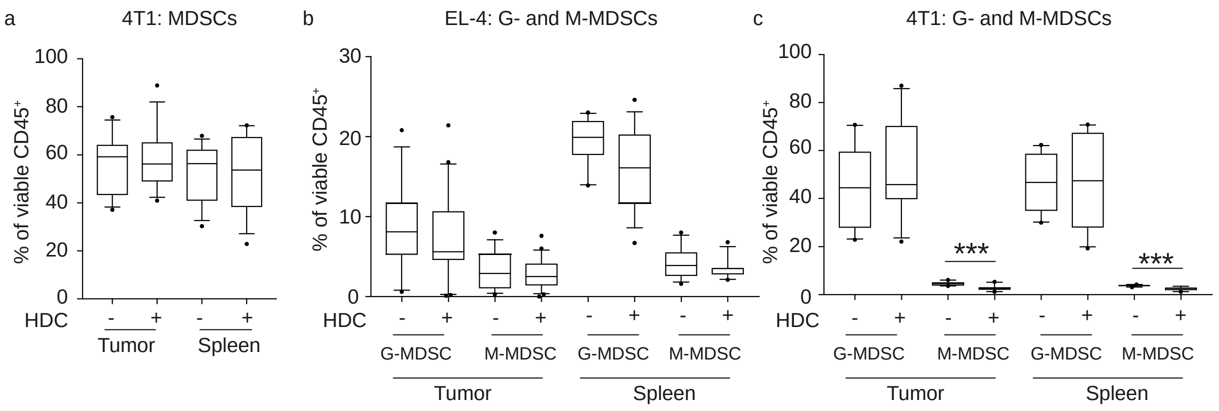

Supplementary figure 3

Effect of HDC on G- and M-MDSCs in EL-4 and 4T1 tumor-bearing mice. (**a**) Accumulation of MDSCs in tumors and spleens of 4T1 tumor-bearing mice (n=15 in tumors and spleens). (**b-c**) Accumulation of tumor and splenic G- and M-MDSCs in (**b**) EL-4 and (**c**) 4T1 tumor-bearing mice. EL-4; intratumoral control: n=19, intratumoral HDC n=21, splenic control: n=12, splenic HDC n=14. 4T1; n=10 for all MDSC populations in both tumor and spleen tissue. Student's *t* test. *** $p<0.001$

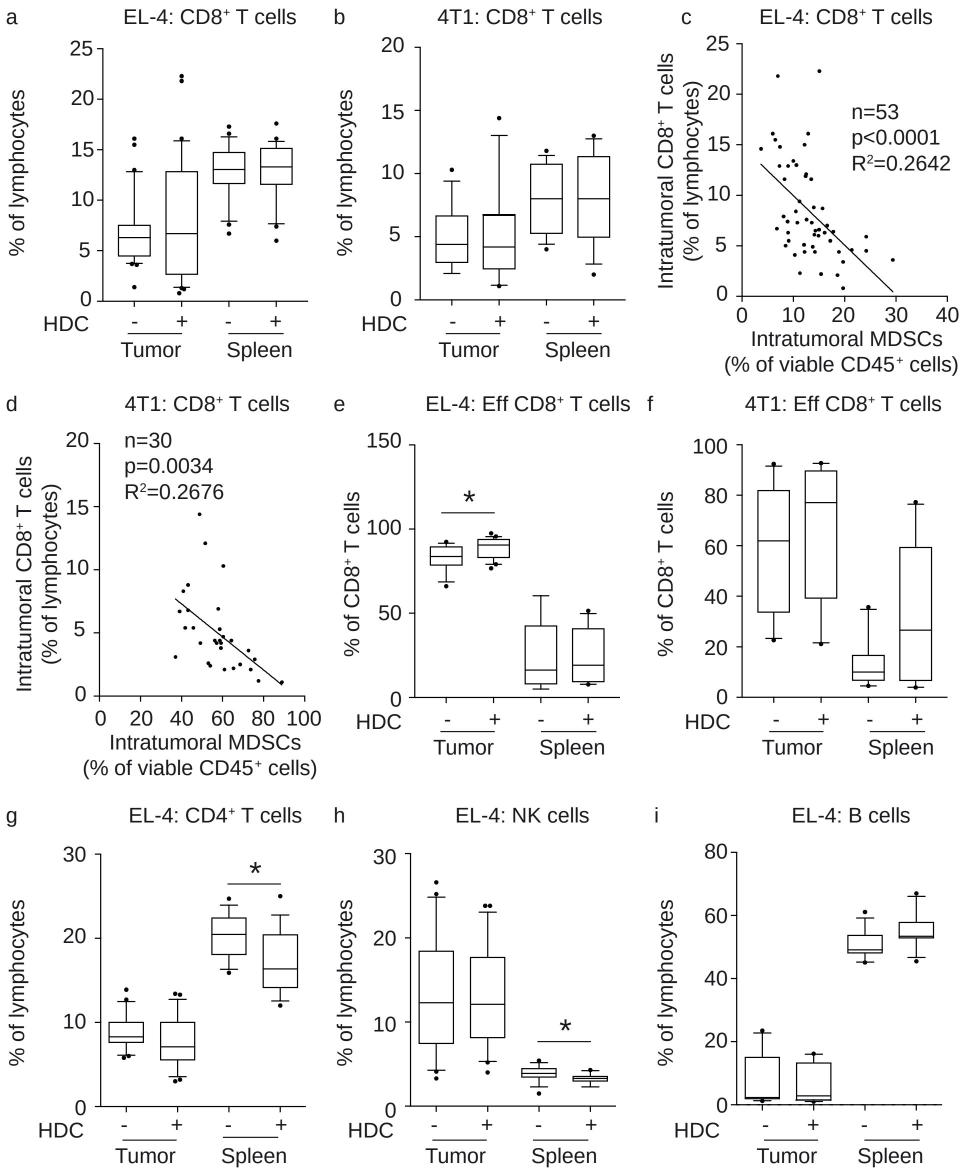

Supplementary figure 4

Effect of HDC on lymphocyte subsets in EL-4 and 4T1 tumor-bearing mice. In mice with (**a**) EL-4 tumors and (**b**) 4T1 tumors the percentage of tumor-infiltrating and splenic $CD8^+$ T cells was determined in control mice (EL-4; n=31 intratumoral, n=22 splenic. 4T1; n=15 for intratumoral and splenic) and in HDC-treated mice (EL-4; n=31 intratumoral, n=23 splenic. 4T1; n=15 for intratumoral and splenic). (**c-d**) Correlation between the fraction of intratumoral MDSCs and tumor-infiltrating $CD8^+$ T cells in control and in HDC-treated (**c**) EL-4 tumor-bearing mice and

(**d**) 4T1 tumor-bearing mice. Panels (**e-f**) show the distribution of T_{eff} cell populations in ctrl and HDC treated (**e**) EL-4-bearing mice (n=18 intratumoral ctrl, n=20 intratumoral HDC, n=9 splenic ctrl, n=11 splenic HDC) and (**f**) 4T1-bearing mice (n=10 for all populations). (**g**) Tumor-infiltrating and splenic $CD4^+$ T cells in control (tumor n=20, spleen; n=16) and HDC-treated (tumor n=22, spleen n=18) EL-4-bearing mice. (**h**) Tumor-infiltrating and splenic NK cells in control (tumor n=22, spleen n=17) and HDC-treated (tumor n=22, spleen n=18) EL-4 -bearing mice. (**i**) Tumor-infiltrating and splenic B cells in control (tumor n=12, spleen n=12) and HDC-treated (tumor n=11, spleen n=12) mice EL-4 -bearing mice. Student's *t* test was used to compare differences between control and HDC-treated mice and linear regression was utilized to calculate correlations. * $p<0.05$

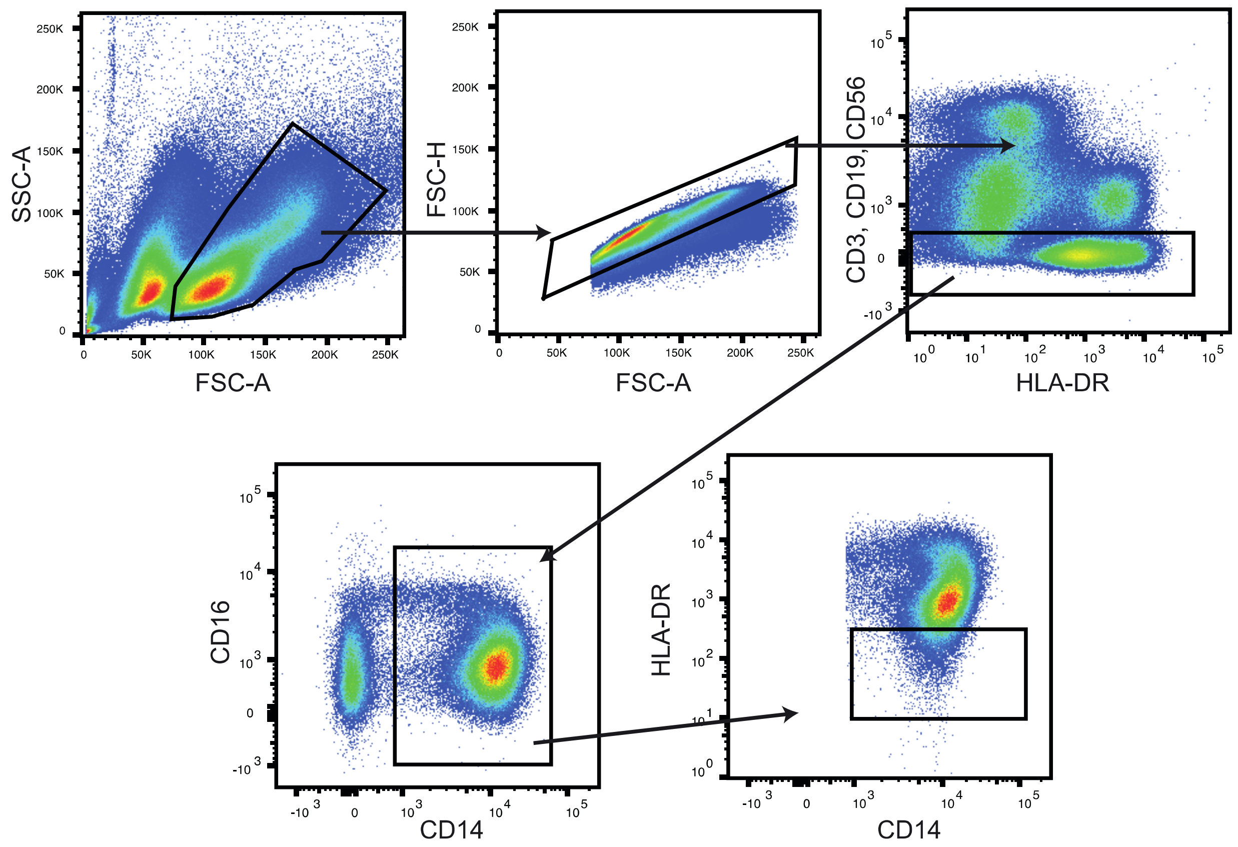

Supplementary figure 5

Gating strategy of M-MDSCs in AML samples. Cells with a morphology of viable cells were gated on a forward-side scatter plot followed by exclusion of duplicates on a FCS-H/FCS-A plot. The $CD3^{+}$, $CD19^{+}$ and $CD56^{+}$ cells were then excluded and among the myeloid cells M-MDSCs were identified as $CD14^{+}HLA\text{-}DR^{low/-}$ cells.

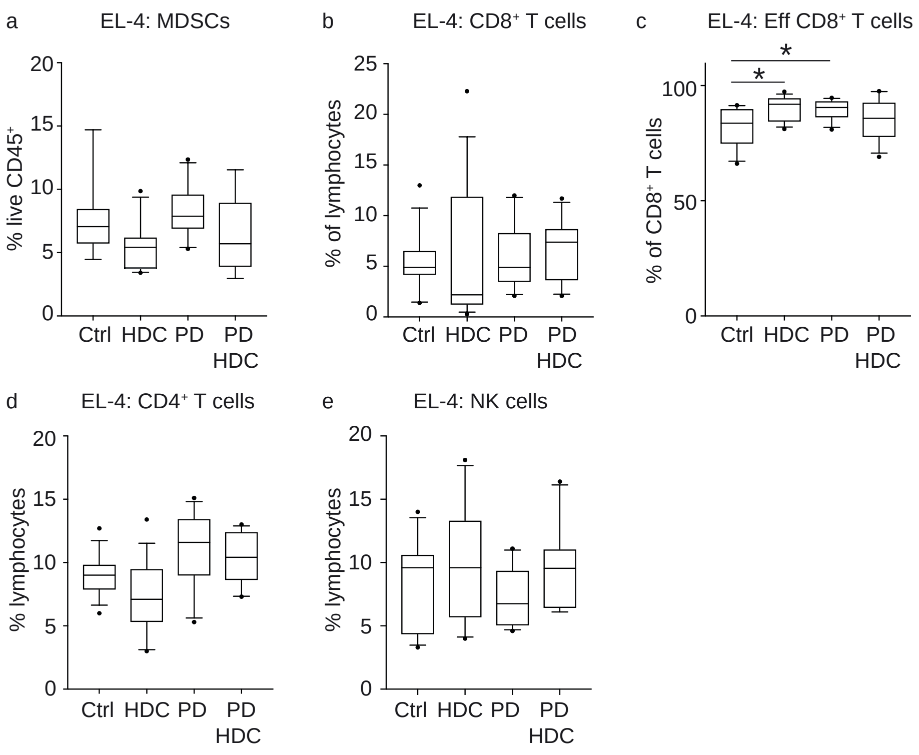

Supplementary figure 6

Effect of α-PD-1/α-PD-L1 and HDC on lymphocyte infiltration in EL-4 tumors. (**a**) Intratumoral accumulation of MDSCs in ctrl (n=9), HDC-treated (n=11), α-PD-1/α-PD-L1-treated (PD, n=10), and HDC/α-PD-1/α-PD-L1 (PD HDC, n=7) mice. Accumulation of (**b**) $CD8^+$ T cells, (**c**) effector $CD8^+$ T cells, (**d**) $CD4^+$ T cells, and (**e**) NK cells in ctrl (n=13), HDC-treated (n=15), α-PD-1/α-PD-L1-treated (PD, n=13), and HDC/α-PD-1/α-PD-L1 (PD HDC, n=11) mice. Analysis by one-way ANOVA

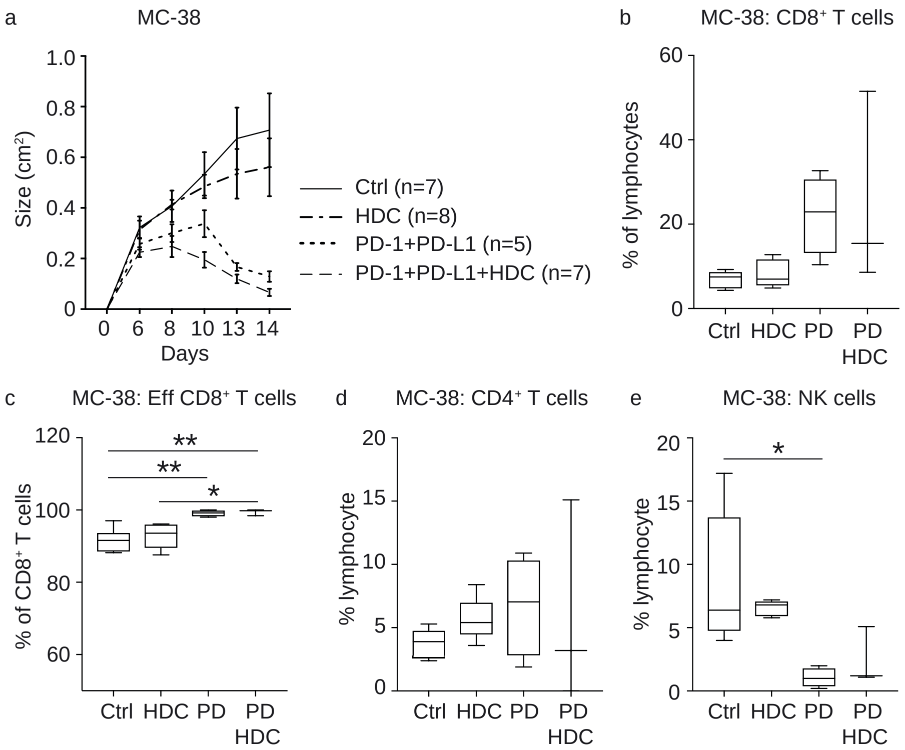

Supplementary figure 7

Effect of α-PD-1/α-PD-L1 and HDC on growth and lymphocyte infiltration in MC-38 tumors. (**a**) growth of MC-38 tumors in control (ctrl, solid line), HDC-treated (dashed-dotted line), α-PD-1/α-PD-L1-treated (dotted line) or HDC/α-PD-1/α-PD-L1-treated (dashed line). Accumulation of (**b**) intratumoral $CD8^+$ T cells, (**c**) effector $CD8^+$ T cells, (**d**) $CD4^+$ T cells, and (**e**) NK cells in control (n=6), HDC-treated (n=5), α-PD-1/α-PD-L1-treated (n=4), and HDC/α-PD-1/α-PD-L1-treated (n=3). Analysis were performed by one-way ANOVA
